# Cost utility analysis of the SQ^®^ HDM SLIT-tablet in house dust mite allergic asthma patients in a German setting

**DOI:** 10.1186/s13601-016-0127-6

**Published:** 2016-09-07

**Authors:** J. Hahn-Pedersen, M. Worm, W. Green, J. Nørgaard Andreasen, M. Taylor

**Affiliations:** 1 ALK, Hørsholm, Denmark; 2 Clinic for Dermatology, Venereology and Allergology, Universitätsmedizin Berlin, Berlin, Germany; 3 York Health Economics Consortium, University of York, York, UK

**Keywords:** House dust mites, Allergic asthma, Cost-utility analysis, Acarizax, Allergy immunotherapy

## Abstract

**Background:**

Asthma affects an estimated 300 million people worldwide with the condition associated with significant healthcare utilisation costs and a large impact on patient quality of life. The SQ^®^ HDM SLIT-tablet (ACARIZAX^®^, Hørsholm, Denmark) is a sublingually administered allergy immunotherapy tablet for house dust mite allergic asthma and allergic rhinitis and has recently been licensed in Europe.

**Objective:**

To assess the cost-effectiveness of ACARIZAX plus pharmacotherapy versus placebo plus pharmacotherapy in patients with house dust mite allergic asthma that is uncontrolled by inhaled corticosteroids, in a German setting. Eligible patients should also have symptoms of mild to severe allergic rhinitis.

**Methods:**

A cost utility analysis was undertaken, based on the results of a European phase III randomised controlled trial, in which ACARIZAX was compared with placebo with both treatment groups also receiving pharmacotherapy in the form of inhaled corticosteroids and short-acting β2-agonists. Cost and quality-adjusted life years from the trial were extrapolated over a nine year time horizon and the incremental cost-effectiveness ratio calculated to compare treatment options.

**Results:**

ACARIZAX plus pharmacotherapy was estimated to generate 6.16 quality-adjusted life years per patient at a cost of €5658, compared with 5.50 quality-adjusted life years (QALYs) at a cost of €2985 for placebo plus pharmacotherapy. This equated to an incremental cost of €2673, incremental QALYs of 0.66 and an incremental cost-effectiveness ratio (ICER) of €4041. The ICER was, therefore, substantially lower than the €40,000 willingness-to-pay threshold per QALY adopted for the analysis. Deterministic sensitivity analyses indicate the results are most sensitive to the utility score of ACARIZAX patients during years 2 and 3 of treatment.

**Conclusion:**

This analysis indicates that ACARIZAX plus pharmacotherapy is cost-effective compared with placebo plus pharmacotherapy for house dust mite allergic asthma patients in Germany. If a disease-modifying effect can be proven the results of this analysis may underestimate the true benefits of ACARIZAX.

## Background

Asthma is a chronic global health problem having a significantly detrimental effect on quality of life and is often associated with significant healthcare utilisation costs. It is estimated that asthma affects 30 million people in Europe and up to 300 million people worldwide, with prevalence expected to rise [1, 2]. In Germany, asthma has been estimated to affect approximately 7 % of the population [3]. Most asthma cases are due to allergic conditions with previous research showing that over 90 % of all allergic asthma patients display symptoms that correlate with allergic rhinitis [4].

Although the disease does not exhibit high mortality rates, the loss in quality of life is significant, with both physical and psychological dimensions found to be affected [5, 6]. It is also associated with substantial costs to the healthcare system and wider society. One European-wide cost-of-illness study found that the mean annual costs of asthma in 2010, including both direct and indirect costs, were €509 and €2281 for controlled and uncontrolled asthma patients respectively [7]. The total asthma burden in Germany was estimated to be €3.3 billion in 2008 [3].

The SQ^®^ HDM SLIT-tablet (ACARIZAX^®^, ALK, Hørsholm, Denmark) is a recently developed allergy immunotherapy (AIT) tablet, targeted specifically at house dust mite (HDM) allergens. It is a sublingual treatment option for HDM allergic asthma patients already taking pharmacotherapy whose symptoms are not well controlled, and is a 1:1 mixture of allergen extract from *Dermatophagoides pteronyssinus* and *Dermatophagoides farinae* [8]. The objective of this analysis was to assess the cost-effectiveness of ACARIZAX from a German societal perspective.

## Methods

### Design

A cost-utility analysis was undertaken based on the results of a double-blinded phase III randomised controlled trial. The MT-04 trial was conducted across 13 European countries, with the primary objective of comparing the efficacy of ACARIZAX with placebo in patients with HDM allergic asthma, as measured by a reduction in the risk of asthma exacerbation. Eligible patients were adults, sensitised to HDM and not well controlled by inhaled corticosteroids (ICS; equivalent to budesonide, 400–1200 μg) at inclusion. Furthermore, they could have multiple sensitizations but a relevant clinical history of perennial allergic asthma or rhinitis caused by other allergens to which the patients were regularly exposed led to exclusion as this may have caused symptoms to arise in the efficacy period that would interfere with the trial results [9]. Patient characteristics are summarised in Table 1. Two treatment groups (6 SQ-HDM and 12 SQ-HDM), and one control group (placebo) were included in the trial to investigate different therapy doses, with the 12 SQ-HDM group considered here. Patient diagnosis took place during the trial screening period. Following screening there was a 7–13 month treatment maintenance period, in which patients in both arms were the given their allocated treatment plus pharmacotherapy in the form of ICS and short-acting β2-agonist (SABA). Patients could also be given oral steroids to treat severe acute asthma symptoms or to restrict the deterioration of asthma symptoms. Following the treatment maintenance period, the daily ICS dose was reduced by 50 % for 3 months and then removed for patients who did not experience an asthma exacerbation. The treatment maintenance period was adopted for the analysis as it was judged to be better aligned with present clinical practice. A more comprehensive description of the trial design has been published previously [9]. Based on the setup of MT-04, two treatment options have been incorporated into the cost-utility analysis: ACARIZAX plus pharmacotherapy (ACARIZAX henceforth) and placebo plus pharmacotherapy (pharmacotherapy henceforth).

**Table 1:** Summary of patient characteristics from MT-04

	Placebo	ACARIZAX
No. of subjects	277	282
Sex (%)		
Male	151 (55 %)	147 (52 %)
Female	126 (45 %)	135 (48 %)
Mean age (SD)	33.0 (12.2)	37 (11.6)
Ethnic origin (%)		
Caucasian	273 (99 %)	277 (98 %)
Other	4 (1 %)	5 (2 %)
Mean years with HDM AA (SD)	13.3 (10.6)	12.9 (11.5)
Control level at randomisation (%)^a^		
Controlled	0 (0 %)	0 (0 %)
Partly controlled	200 (72 %)	200 (71 %)
Uncontrolled	77 (28 %)	82 (29 %)

*AA* allergic asthma, *HDM* house dust mite, *SD* standard deviation

^a^Classification system based on GINA, Masoli et al. [2]

### Healthcare resource use

Within MT-04, patients recorded medication use using electronic diaries during the last 4 weeks of the treatment maintenance period. Physician and emergency room visits were also recorded by trial investigators at each visit. In the analysis, all resources recorded within MT-04 were combined with relevant unit costs from a German perspective, to estimate mean patient costs over a one year time horizon. The cost of ACARIZAX was also included, with a patient requiring one tablet per day. ACARIZAX is suitable for home treatment, although the first tablet should be taken under the surveillance of a physician. This additional visit was also incorporated into the model. Healthcare resource use values implemented in the analysis are summarised in Table 2.

**Table 2:** Summary of cost and resource use inputs incorporated in the analysis

Resource	Unit price	Annual resource use		Total cost	
		ACARIZAX	Pharmacotherapy	ACARIZAX	Pharmacotherapy
ACARIZAX tablet^a^	€2.53	365	0	€923	€0
GP visits^b^	€29.35	0.175	0.105	€5.13	€3.07
Emergency room visits^b^	€74.96	0.010	0.025	€0.75	€1.89
ICS daily dose (μg)^c^	€18.14	563	555	€373	€363
SABA intake (doses)^c^	€22.15	266	297	€9.82	€10.96

Three drugs and two other medical resources were included as parameters in the analysis. Resource use was based on data recorded in MT-04 and, therefore, they relate specifically to allergic asthma patients. The values have been multiplied by the unit price of each resource to generate total costs. These costs were applied to a one year period, and applied equally across all years in the analysis (with costs also discounted at 3 % per year in line with German guidelines)

^a^Source: http://www2.lauer-fischer.de/

^b^Source: http://www.kbv.de/html/

^c^Source: https://www.gkv-spitzenverband.de

The analysis was also run with sick days considered, to capture the impact of indirect costs. Within MT-04, the impact of asthma on productivity was captured via the administration of the work productivity and activity impairment (WPAI) questionnaire. The WPAI is a well-validated instrument that measures absenteeism, presenteeism and impairments in unpaid activity over the previous seven day period [10].

### Patient quality of life

The impact of allergic asthma on patient health-related quality of life (HRQoL) was captured. Patient reported health outcomes were used to elicit utility scores; which are the valuing of health on a scale of 0–1, with 0 representing health states equivalent to death and 1 representing full health. Quality-adjusted life years (QALYs) combine utility values and time to determine HRQoL over time. A patient who experiences one year in full health, (i.e. with a utility value of 1), would gain 1 QALY. Similarly, a patient who experiences 2 years with health valued at 0.5 would gain 1 QALY [11]. Utilities have been used to estimate QALYs for patients in both treatment groups.

Utility values used in the model were taken from the end of the treatment maintenance period in MT-04 (i.e. before ICS reduction and removal). Within the trial the SF-36 health survey was used to measure patient utility. For values used within the analysis, the data was corrected for baseline to determine between group differences at the end of the treatment maintenance period. These utility scores are summarised in Table 3.

**Table 3:** Summary of utility values applied to patients in the analysis

Utility at baseline—all patients	Change from baseline			Utility adopted in analysis	
	*Placebo*	ACARIZAX	*p* value	*Placebo*	ACARIZAX
0.736	0.0059	0.0315	0.0318	0.742	0.768

Within MT-04 patient utility was measured using the SF-36 survey instrument. There was a statistically significant difference in utility change (i.e. a measurement of patient quality of life) from baseline to the end of the treatment maintenance period in MT-04 between ACARIZAX and placebo (*p* < 0.05). These values were applied to the mean utility score at baseline for all patients, to estimate the values that were applied at baseline in the analysis. Overall, ACARIZAX patients had a greater quality of life compared to placebo patients, at the end of the treatment maintenance period

### Pharmacoeconomic analysis

To assess the long-term impact of ACARIZAX on the healthcare system and patient HRQoL, costs and QALYs from MT-04 have been extrapolated over a nine-year time horizon. For this extrapolation, costs that occurred during year one are applied equally across all years. To examine the impact of treatment on patient health, QALY scores were altered using an annual rate of change in utility (i.e. quality of life). There is evidence that AIT may have both a curative and preventative impact on respiratory allergies, equating to a long-term treatment effect [12–14]. Evidence specific to GRAZAX^®^ therapy, shows that the treatment has a disease-modifying effect, as patients receive clinical benefit for at least 5 years despite treatment only lasting 3 years [15]. In the analysis it has been assumed that there will be a 5 % increase in utility for ACARIZAX patients during years two and three of treatment, based on the assumption patients will continue to receive a clinical benefit from treatment. Alternatively, for pharmacotherapy patients it is assumed that patient health remains stable, based on the assumption that the improvement that was observed during the trial for pharmacotherapy patients will remain throughout this period. Following the treatment period it is assumed that both patient groups remain stable for years four and five (i.e. 2 years after discontinuation of treatment), followed by a 5 % decline in health during years 6–9.

Based on these assumptions, costs and QALYs have been estimated for both treatment groups over the nine-year time horizon and an assessment of the cost-effectiveness undertaken. Cost-effectiveness was defined using the incremental cost-effectiveness ratio (ICER) of ACARIZAX in addition to pharmacotherapy versus pharmacotherapy alone. To estimate the cost-effectiveness of an intervention to society as a whole, it is necessary to compare the ICER against a willingness-to-pay threshold. This allows the value of one QALY to society to be defined in monetary terms. In Germany, rationing decisions are made by Gemeinsamer Bundesausschuss (G-BA) on a case-by-case basis and, therefore, there is no defined threshold [16]. However in the United Kingdom, the National Institute for Health and Care Excellence (NICE), a world leader in national health technology assessments, uses a threshold of £20,000 to £30,000 per QALY (€27,473 to €41,209) [17]. For an intervention to be cost-effective, the ICER should fall within or below this threshold. For this analysis, a threshold value of €40,000 (~£30,000) has been used. This means that compared with pharmacotherapy only, ACARIZAX should generate one QALY at a cost less than or equal to €40,000.

$${\text{ICER}} = \frac{{Cost_{\text{Treatment}} - Cost_{\text{Comparator}} }}{{{\text{QALY}}_{\text{Treatment}} - {\text{QALY}}_{\text{Comparator}} }} = \frac{{\Delta {\text{Cost}}}}{{\Delta {\text{QALY}}}}$$

Because a nine-year time horizon has been adopted, cost and QALY values were discounted using an annual discount rate of 3 %, in line with German guidelines [18].

### Uncertainty analysis

**Fig. 1:**
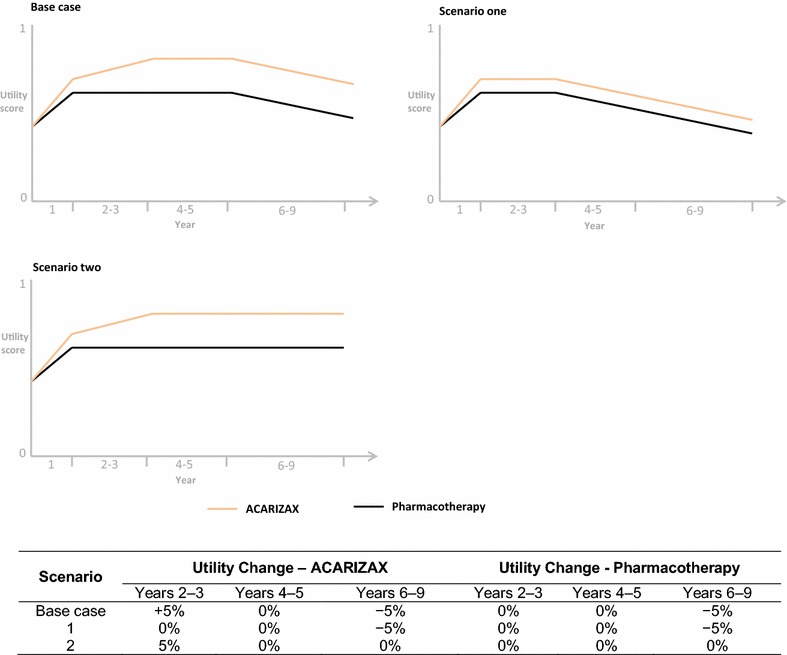
Graphical representation of change in patient utility over time, for each of the three scenarios assessed. Within all three scenarios, utility scores were based on results from the end of the treatment maintenance period in MT-04. For all remaining years, utility scores were altered via an annual rate of change, to see the impact long-term changes in patient outcomes had on the results. The rates of change in each scenario are given here in table

To investigate the impact of changes in long term patient utility, two scenarios have been tested alongside the base case analysis. The rate of utility change for ACARIZAX and pharmacotherapy in the two scenarios are depicted graphically in Fig. 1. In short, within scenario one it is assumed that utilities (i.e. quality of life) remain stable during the treatment period (i.e. years 2–3), followed by a 5 % decline for all subsequent years, for both treatment groups. This is to test the impact of a decline in health as soon as treatment is stopped, an unfavourable scenario. In scenario two it is assumed that ACARIZAX patients have a small 5 % increase in utility for years two and three, followed by stability for all remaining years, whilst the utility for pharmacotherapy remains stable for the full time horizon. This scenario has been implemented to test the impact of a lasting disease-modifying effect for ACARIZAX that leads to long-term improvements in patient health.

In order to account for first-order uncertainty around the data used for input parameter values, one-way deterministic sensitivity has also been undertaken. This involves altering the value used for individual parameters within realistic ranges, to assess the impact on the model’s results.

## Results

**Fig. 2:**
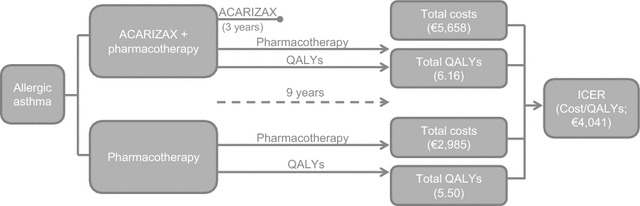
Overview of the structure of the analysis. A cost utility analysis was undertaken to assess the impact of ACARIZAX on allergic asthma patients taking pharmacotherapy. Two treatment options were included; ACARIZAX plus pharmacotherapy, and placebo plus pharmacotherapy. A nine year time horizon was used, with ACARIZAX patients given treatment for 3 years. Over the nine year time horizon ACARIZAX patients accumulated 6.16 QALYs on average, at a mean cost of €5658, compared to an average of 5.50 QALYs at a mean cost of €2985 for pharmacotherapy only patients. This equates to an incremental cost-effectiveness ration (ICER) of €4041, substantially lower than the threshold value of €40,000 adopted here. This indicates that ACARIZAX is a cost-effective treatment option

The overall setup of the analysis and key results are presented in Fig. 2. The results of the base case analysis indicate that, over the nine year time horizon, ACARIZAX in addition to pharmacotherapy leads to a total of 6.16 QALYs per patient at a cost of €5658, compare with 5.50 QALYs at a cost of €2985 for pharmacotherapy alone. Therefore, ACARIZAX produced an extra 0.66 QALYs at an incremental cost of €2673, which equates to an ICER of €4041. This is substantially lower than the €40,000 threshold adopted for the analysis.

Indirect costs were also incorporated based on the administration of the WPAI during the final month of the MT-04 treatment maintenance period. Following this assessment it was found that asthma caused 0.8 and 1.6 % of work time to be missed for ACARIZAX and pharmacotherapy respectively. Based on an average of 1371 h worked per year in Germany [19] and an average work day of 7.5 h, it was estimated that 1.51 days per year would be missed with ACARIZAX compared with 3.02 days with pharmacotherapy. Klussman and colleagues have previously estimated the cost per sick day in Germany is €93.69 [20]. Therefore, the total annual indirect costs were estimated to be €141 for ACARIZAX and €283 for pharmacotherapy. When these costs were included the total per patient costs rose to €6760 for ACARIZAX and €5188 for pharmacotherapy respectively, with overall incremental costs reducing from €2673 to €1572.

Within the two additional scenarios, the ICER remained below €40,000. For scenario one (i.e. the unfavourable scenario) the ICER increased from the base case value to €14,091, as the QALY gain reduced to 0.19. Alternatively, for scenario two (i.e. scenario testing the disease-modifying effect) the ICER was lower than that produced in the base case, being as it was €3832, and this was due to a QALY gain of 0.70. Within both scenarios, the inclusion of indirect costs had no impact on the direction of the results but did affect the magnitude, with the incremental costs reducing.

The results of the sensitivity analyses indicate that the model’s results are sensitive to changes in two key input parameters. If, during years two and three of treatment, ACARIZAX patients have a decline in health of 2 % or more, or if pharmacotherapy patients have an improvement in health of 5 % or more during years 6–9, then the ICER increases above the threshold value of €40,000. For all remaining parameters, the changes had no impact on the model’s conclusions.

## Discussion

The cost-utility analysis illustrates that, over a nine year time horizon, ACARIZAX plus pharmacotherapy is cost-effective compared with placebo plus pharmacotherapy, in HDM allergic asthma patients not well controlled by ICS and associated with mild to severe allergic rhinitis in a German setting. The deterministic sensitivity analyses show that there is a degree of uncertainty regarding the results of the analysis, given modest changes in two input parameters impacted on the cost-effectiveness of ACARIZAX (as measured by the ICER). If patient health declines at a rate of 2 % or higher during the 3 years of treatment with ACARIZAX, then it can no longer be considered a cost-effective treatment option at the given threshold value. However, evidence regarding AITs indicates that the efficacy of these treatments may be sustained for the duration of treatment, and beyond [12–15]. Therefore, it is unlikely that the health of ACARIZAX patients will decline during the treatment period. Similarly, if patient health with pharmacotherapy improves during years 6–9 then ACARIZAX is no longer cost effective. However, this improvement is unlikely given the applicable patient population, as they are patients whose symptoms are uncontrolled by pharmacotherapy.

The analysis was conducted based on the results of a single RCT and there are certain limitations with this approach. In particular, healthcare utilisation is based on resource use data collected solely within MT-04, with costs specific to Germany applied to this data. This means the values used in the analysis may not be reflective of clinical practice, as the resource use within the trial was protocol driven, and certain resources required by allergic asthma patients may not have been captured as they were not recorded in the trial. For example, pharmacotherapy patients received more clinical supervision than can be expected in clinical practice. This may have reduced the total number of contacts with the healthcare system outside of the trial protocol (e.g. emergency room visits). Related to this, better overall supervision and the Hawthorne effect (i.e. patients modifying their behaviour as they were being monitored) may have led to a clinical gain that will not be found in practice, and patient health may have improved regardless of treatment as outcome measures naturally tended towards the population mean (i.e. regression to the mean). Despite the limitations of undertaking economic evaluations alongside a single RCT such an approach is becoming more common, with some funders now specifically requesting these evaluations alongside RCTs, as they allow for an early stage estimate of cost-effectiveness (i.e. before use has become widespread in clinical practice) [22]. Furthermore, MT-04 was a large-scale trial conducted across several countries with multiple investigators. Therefore, the treatment effect of ACARIZAX should have been appropriately captured across a wide population group.

The analysis covers a nine year time horizon with the assumption that the health (i.e. utility) of ACARIZAX patients remains stable in the 2 years post-treatment, which implies some form of disease-modifying effect. However, there are no long-term efficacy data to prove this effect, with the assumption based on the findings of other AITs. Therefore, to ensure that ACARIZAX does not gain an unfair advantage, it was also assumed that pharmacotherapy patients remained stable during this period and in the longer term that both ACARIZAX and pharmacotherapy patients have a decline in health during years 6–9. Furthermore, at the willingness-to-pay threshold of €40,000, ACARIZAX was cost-effective compared with pharmacotherapy at each of the 9 years included in the analysis indicating it is cost-effective even when long-term extrapolation of the data is not undertaken. In the future, further health economic evaluations could be undertaken should long-term, real world data become available (e.g. registry studies). This evaluation could estimate the true benefits of ACARIZAX, particularly if a disease-modifying effect has been proven in practice, and the impact of the placebo effect observed in MT-04 could also be assessed. If a disease-modifying effect can be proven in practice then this analysis may prove to be conservative estimation of the cost-effectiveness of ACARIZAX.

AIT options, such as ACARIZAX, can be associated with adverse events that will impact on patient quality of life and overall treatment costs. Such events were not formally included in this analysis as the rate of serious adverse events was very low (less than 1 % for 12 SQ HDM patients) in MT-04 and because there was no clear difference in the rate of serious adverse events between ACARIZAX and placebo patients. It should be noted that the cost of asthma exacerbations were also not included in the analysis. However, ACARIZAX has a positive impact on exacerbations as shown via the quantification of number needed to treat (NNT) to avoid any moderate to severe exacerbation, which was measured during the ICS reduction period of trial, and found to only be 10 for ACARIZAX [21]. This beneficial impact of ACARIZAX was not quantified in the analysis.

In the future it may be pertinent to compare ACARIZAX with other AITs, which are considered standard care in Germany. It was, however, not feasible to compare ACARIZAX with all potential AITs in a double-blinded, controlled trial. Furthermore, currently, there are no other AITs for which the efficacy in (HDM) allergic asthma has been demonstrated using similar design; hence comparison between ACARIZAX and other AIT is not possible [23].

## Conclusion

The analysis presented here indicates that ACARIZAX in addition to pharmacotherapy is a cost-effective treatment option compared to pharmacotherapy alone in HDM allergic asthma patients not well controlled by ICS and associated with mild to severe allergic rhinitis. If a disease-modifying effect can be proven the results of this analysis may underestimate the true benefits of ACARIZAX as conservative assumptions were used to predict long-term patient outcomes.

## Abbreviations

AIT: allergy immunotherapy
G-BA: Gemeinsamer Bundesausschuss
GP: general practice
HDM: house dust mite
HRQoL: health-related quality of life
ICER: incremental cost-effectiveness ratio
ICS: inhaled corticosteroids
NICE: national institute for health and care excellence
QALY: quality-adjusted life year
SABA: short-acting β2-agonist
SF-36: 36 item short form health survey
WPAI: work productivity and activity impairment
